# Phosphorylation of the Chaperone-Like HspB5 Rescues Trafficking and Function of F508del-CFTR

**DOI:** 10.3390/ijms21144844

**Published:** 2020-07-08

**Authors:** Fanny Degrugillier, Abdel Aissat, Virginie Prulière-Escabasse, Lucie Bizard, Benjamin Simonneau, Xavier Decrouy, Chong Jiang, Daniela Rotin, Pascale Fanen, Stéphanie Simon

**Affiliations:** 1 Univ Paris Est Creteil, INSERM, IMRB, F-94010 Creteil, France; fanny.degrugillier@inserm.fr (F.D.); abdel.aissat@inserm.fr (A.A.); virginie.pruliere@inserm.fr (V.P.-E.); lucie.bizard@chicreteil.fr (L.B.); benjamin.simonneau@inserm.fr (B.S.); xavier.decrouy@inserm.fr (X.D.); pascale.fanen@inserm.fr (P.F.); 2 AP-HP, Hôpital Henri Mondor, Département de Génétique, F-94010 Creteil, France; 3 Centre Hospitalier Intercommunal de Creteil, Service d’ORL et de Chirurgie Cervico-Faciale, F-94010 Creteil, France; 4 The Hospital for Sick Children and the University of Toronto, Toronto, ON M5G 0A4, Canada; chong.jiang@utoronto.ca (C.J.); drotin@sickkids.ca (D.R.)

**Keywords:** cystic fibrosis, CFTR, HspB5, alpha B-crystallin, CRYAB, phosphorylation

## Abstract

Cystic Fibrosis is a lethal monogenic autosomal recessive disease linked to mutations in Cystic Fibrosis Transmembrane Conductance Regulator (CFTR) protein. The most frequent mutation is the deletion of phenylalanine at position 508 of the protein. This F508del-CFTR mutation leads to misfolded protein that is detected by the quality control machinery within the endoplasmic reticulum and targeted for destruction by the proteasome. Modulating quality control proteins as molecular chaperones is a promising strategy for attenuating the degradation and stabilizing the mutant CFTR at the plasma membrane. Among the molecular chaperones, the small heat shock protein HspB1 and HspB4 were shown to promote degradation of F508del-CFTR. Here, we investigated the impact of HspB5 expression and phosphorylation on transport to the plasma membrane, function and stability of F508del-CFTR. We show that a phosphomimetic form of HspB5 increases the transport to the plasma membrane, function and stability of F508del-CFTR. These activities are further enhanced in presence of therapeutic drugs currently used for the treatment of cystic fibrosis (VX-770/Ivacaftor, VX-770+VX-809/Orkambi). Overall, this study highlights the beneficial effects of a phosphorylated form of HspB5 on F508del-CFTR rescue and its therapeutic potential in cystic fibrosis.

## 1. Introduction

Newly synthesized proteins undergo stringent quality control (QC). During biogenesis, to exit from the endoplasmic reticulum (ER), proteins must typically reach a native conformation that corresponds to the most energetically favorable state. If the folding process and maturation fail, proteins identified by the QC are not transported to their final destination in cell and are instead directed to the degradation machinery called the ER-associated degradation process (ERAD). Because they interact specifically with improperly folded proteins, molecular chaperones are major sensors, able to assist the folding mechanism but also to dispatch any improperly folded proteins for destruction [1]. In particular, transmembrane proteins (TMPs) must overcome huge energy barriers to reach their native conformation linked to their complex structure and topology [2]. Numerous human hereditary pathologies are related to the loss of function of mutated TMPs (receptors, channels, pumps, or transporters), which then fail to attain their native conformation [3]. In some cases, the mutation primarily affects the transport of the TMPs but does not completely abolish their biological activity. This phenomenon is particularly well illustrated by some mutations in the Cystic Fibrosis Transmembrane conductance Regulator (CFTR) protein, responsible for Cystic Fibrosis (CF). Among the Caucasian population, CF is the most common lethal monogenic autosomal recessive disease. CFTR normally functions as a chloride/bicarbonate channel at the epithelial cells’ plasma membrane (PM) [4]. The most frequent mutation, a deletion of phenylalanine 508 (F508del-CFTR), is present in approximately 85% of CF patients. The F508del mutation induces CFTR misfolding, targeting 99% of the mutated protein to ERAD [5]. Different checkpoints have been proposed to drive CFTR exit from the ER: ‘folding’ checkpoints involve mainly interaction with molecular chaperones, whereas ‘trafficking’ checkpoints involve recognition of trafficking/exit signals leading to the incorporation of the proteins into vesicles [6]. It is now well established that modulating the chaperone activity would be a viable target to limit the degradation and/or to stabilize F508del-CFTR at the PM, therefore increasing its function [7]. Many studies have shown crucial and dual functions of the core molecular chaperones, the heat shock proteins (Hsps), especially the Hsp90, Hsp70, and Hsp40 families, on the biogenesis of wild-type (WT)- or F508del-CFTR [8,9,10,11]. Most recently, two members of small Hsps (sHsps), HspB1 (Hsp27) and HspB4 (αA-crystallin, CRYAA), were identified in pathways that promote selective degradation of F508del-CFTR with a modest effect on the WT protein [12,13]. Interestingly, compounds able to correct PM localization (correctors) partly disrupt the binding to HspB1 of F508del-CFTR or CFTR mutated within the Nucleotide-Binding Domain 1 [14,15]. Indeed, HspB1 reduction by siRNA rescued F508del-CFTR, however this rescue was not synergistic or additive with the treatment by correctors, suggesting a common pathway between these molecules and the network of ERAD involving HspB1 [14]. These observations prompted us to evaluate the impact of another sHsp, HspB5 (αB-crystallin, CRYAB). Originally discovered as a structural protein in ocular lens, HspB5 is also present in number of nonlenticular tissues, strongly in those with high levels of oxidative function but also less abundantly in other tissues, including the lung [16,17]. HspB5 counteracts aggregation and rescues proper localization of mutated Frizzled 4 receptor and ATP7B, two mutated TMPs associated, respectively, with familial exudative vitreoretinopathy and Wilson’s disease [18]. This beneficial effect on the misfolded TMPs was demonstrated to be regulated by HspB5 phosphorylation status [19]. In view of these data, we decided to evaluate if HspB5 could facilitate misfolded CFTR degradation, much like HspB1 and HspB4, or in contrast, promotes proper localization of mutated CFTR, as reported with other TMPs.

Here, we identified one phosphomimetic form of HspB5 having a beneficial effect on transport and function of F508del-CFTR. These activities are further enhanced in the presence of drugs used for the treatment of CF. Overall, this study uncovers a novel role for HspB5 (compared to HspB1 and HspB4) in CF.

## 2. Results

### 2.1. Endogenous Expression of HspB5 Protein in Different Cystic Fibrosis Models

**Figure 1:**
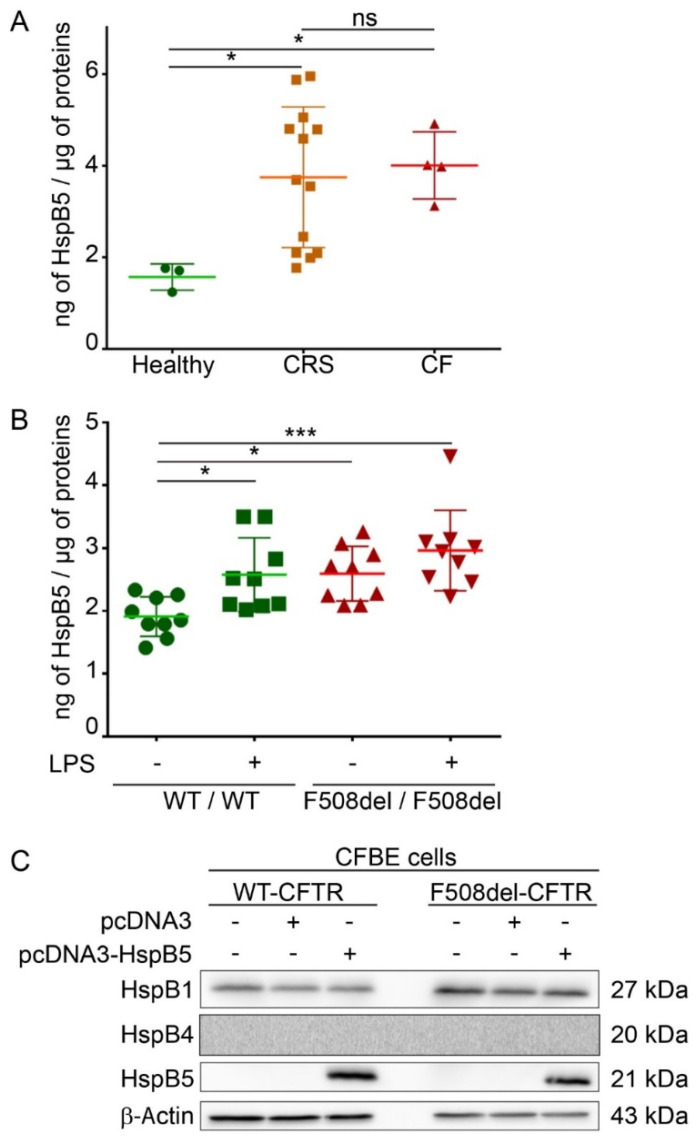
Endogenous expression of HspB5 in different Cystic Fibrosis (CF) models. Measurement of the heat shock (HspB5) protein level was done by ELISA on total protein extracts. (**A**) HspB5 protein content in human epithelial nasal cells (HNEC) cultivated in an air–liquid interface from nasal brushing of healthy subjects (*n* = 3) or polyps from patients with CF (*n* = 4) or with chronic rhinosinusitis (CRS) (*n* = 21). * *p* < 0.05 compared to controls; ns = non-significant. Differences were obtained using a Kruskal–Wallis test. (**B**) HspB5 protein content in the lung of mice homozygous for the F508del-Cystic Fibrosis Transmembrane Conductance Regulator (CFTR) mutation (F508del/F508del) and normal homozygous wild-type (WT) littermates (+/+) 3 h following intratracheal instillation of 400 μg·kg^−1^ lipopolysaccharides from *P. aeruginosa* (LPS) or an equivalent volume of saline (Veh.) (*n* = 3, measurements in triplicate). * *p* < 0.05 and *** *p* < 0.001 compared to (+/+) mice with vehicle. Differences were obtained using a one-way ANOVA followed by the post-hoc Dunnett’s test. (**C**) Human CF bronchial epithelial cells (CFBE) stably expressing WT- or F508del-CFTR were transfected, or not, with empty vector or HspB5 construct. Cells were harvested 24 h after transfection and processed for SDS-PAGE/Western blotting using anti-HspB1, -HspB4, or -HspB5 antibodies. Equal loading was verified using anti-β-Actin antibody. Untransfected cells were used as a negative control. Representative images are shown (*n* = 3).

HspB5 is described as a strongly expressed protein in cells that have high levels of oxidative function [17] but also weakly in different types of epithelial cells and lung tissue [16]. A reliable *in vitro* CF model is human epithelial nasal cells (HNEC) cultured from CF patients. These cultured cells grown at an air–liquid interface allow in vitro prediction of respiratory improvement in CF patients treated with CFTR modulators [20]. HNEC were here obtained from nasal polyp surgery of CF patients (*n* = 4), from patients with chronic rhinosinusitis (CRS) (*n* = 13), or from nasal brushing of healthy subjects as control (*n* = 3). To determine if HspB5 is expressed in HNEC, we performed an ELISA assay on total protein extracts. Our results showed that HspB5 is weakly expressed in HNEC of healthy subjects (1.57 ± 0.22 ng/µg of proteins) but more strongly expressed in HNEC derived from nasal polyps from patients with CF (4 ± 0.45 ng/µg of proteins) or with CRS (3.74 ± 1.31 ng/µg of proteins) (Figure 1A). We confirmed this result in lung of CF mice homozygous for the F508del-CFTR mutation (F508del/F508del) (2.59 ± 0.37 ng/µg of proteins) compared to the WT (+/+) mice (1.91 ± 0.25 ng/µg of proteins) (*n* = 3, measurements in triplicate) (Figure 1B). Interestingly, after intratracheal instillation of lipopolysaccharides from *P. aeruginosa* to promote lung inflammation (*n* = 3), a significant increase in the HspB5 level was observed in (+/+) mice (2.57 ± 0.47 ng/µg of proteins), whereas no significant change was observed in F508del/F508del mice (2.96 ± 0.41 ng/µg of proteins) (Figure 1B). To validate another model in which we could recover enough quantity of proteins suitable in various tests, we used two human bronchial epithelial cell lines: WT- and F508del-CFTR CFBE cells. In these cells, we checked the level of endogenous HspB5 and the two other sHsps previously studied in CF (HspB1 and HspB4), by immunoblot. Our data revealed that, whereas HspB1 is expressed endogenously in CFBE cell lines (WT- and F508del-CFTR), HspB4 and HspB5 were not (Figure 1C). Moreover, transient expression of HspB5 was homogeneous (Supplemental Figure S1A) and did not induce a change in HspB1 and HspB4 endogenous expression level (Supplemental Figure S1B). The absence of HspB4 expression was further confirmed by visualization of a signal in transfected cells to control the antibody (Supplemental Figure S1C). These data corroborate our choice of this cellular model to decipher the impact of HspB5 expression in CF.

### 2.2. Phosphorylation Pattern of Overexpressed HspB5 is Altered in F508del-CFBE Cell Line Compared to WT-CFBE Cell Line

**Figure 2:**
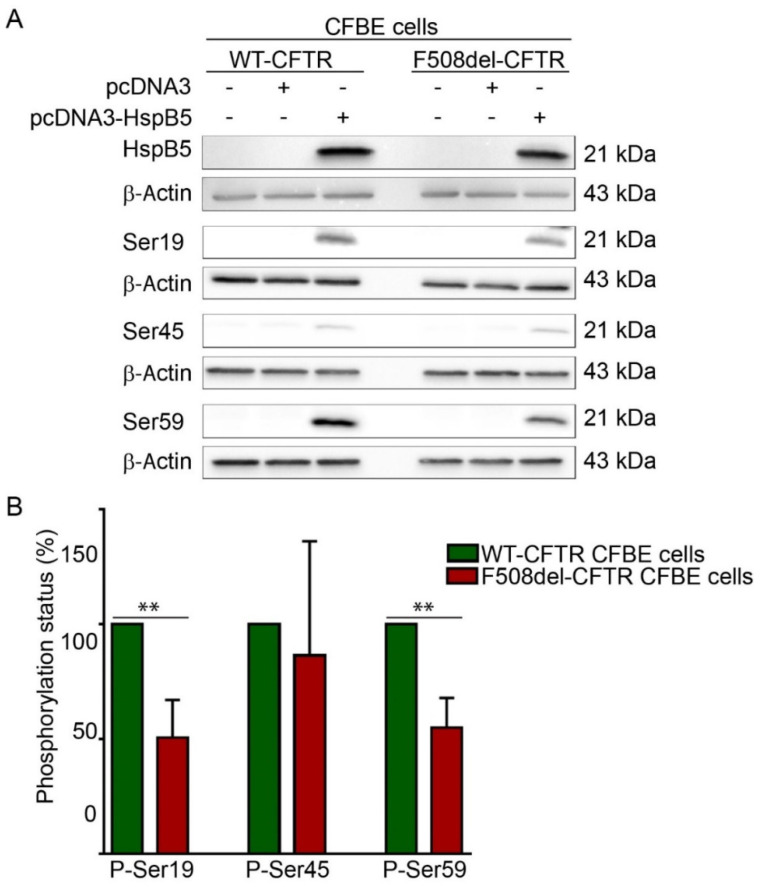
Determination of HspB5 phosphorylation status. (**A**) Western blotting using the phospho-specific anti-Ser19, -Ser45, and -Ser59 antibodies. Equal loading and protein expression were verified using anti-β-Actin and anti-HspB5 antibodies. Untransfected cells were used as a negative control. Representative images are shown (*n* = 5). (**B**) Quantification of the involvement of the single phosphorylation sites of HspB5, as shown in A. Quantification was done using Gene Tools software. The degree of phosphorylation of the various HspB5 species was determined as previously [21]. ** *p* < 0.01 compared to signal of HspB5 in WT-CFTR CFBE. Differences were obtained using a one-way ANOVA followed by the post-hoc Dunnett test.

The ability of HspB5 to correct localization of TMPs at the PM was suggested to be dependent on phosphorylation [19]. HspB5 phosphorylation status was assessed using phosphoserine-specific antibodies that recognize the three known phosphoserines, Ser-19, Ser-45, and Ser-59 in HspB5. These experiments were performed in transiently transfected WT- or F508del-CFTR CFBE cell extracts (Figure 2A). Phosphorylation was detected on the three HspB5 phosphorylation serine sites in both WT- and F508del-CFTR CFBE cells. Interestingly, HspB5 was hypophosphorylated on Ser-19 and Ser-59 in F508del-CFTR CFBE as compared to CFTR-WT CFBE cells, whereas no significant change was observed on Ser-45 (Figure 2B). In conclusion, an abnormal degree of phosphorylation (hypophosphorylation) was detected in CFTR-F508del CFBE as compared to CFTR-WT CFBE cells.

### 2.3. Phosphorylation-Dependent Ability of HspB5 to Rescue the Plasma Membrane Localization of F508del-CFTR

**Figure 3:**
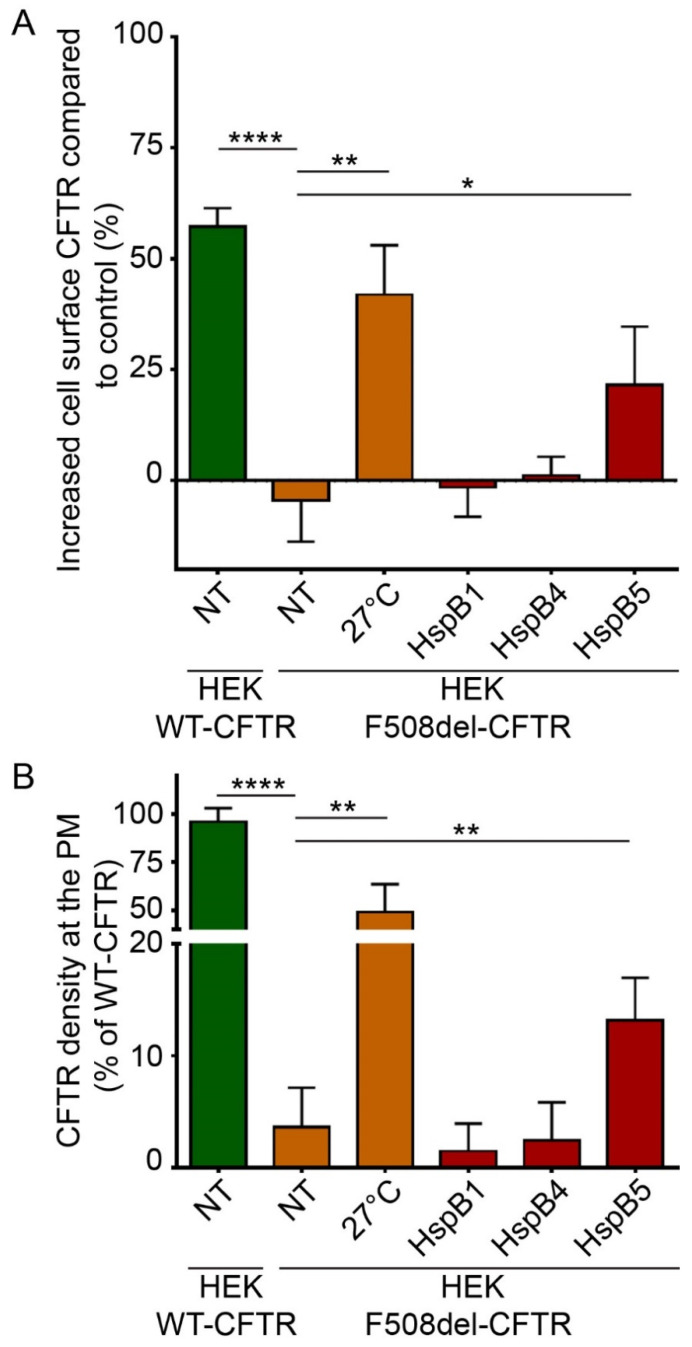
Impact of overexpression of HspB1, HspB4, or HspB5 on localization at the plasma membrane (PM) of F508del-CFTR in human embryonic kidney HEK293 GripTite (HEK) cells. HEK cells stably expressing F508del-CFTR-3HA were transfected with a HspB1, HspB4, or HspB5 construct. Cells were used 24 h post-transfection for detection of PM CFTR (*n* = 5). Untransfected F508del-CFTR-3HA HEK (NT) were used as a negative control. Untransfected F508del-CFTR-3HA HEK cultivated at 27 °C (27 °C), and untransfected WT-CFTR-3HA HEK (NT) were used as positive controls. * *p* < 0.05, ** *p* < 0.01, **** *p* < 0.0001 compared to untransfected F508del-CFTR-3HA HEK (NT). Differences were obtained using a one-way ANOVA followed by the post-hoc Dunnett test. (**A**) ELISA-based assay to measure CFTR at the PM. Each condition was analyzed in duplicate using 5 µg of protein extracts. Experiments were conducted five times. (**B**) Cell surface density measurement. Each condition was analyzed in duplicate. Experiments were conducted five times.

To evaluate and quantify the PM localization of WT- and F508del-CFTR, we used two different ELISA-based methods. These methods are more robust and sensitive than quantification of C band of F508del-CFTR by immunoblotting, which reveals only the fully-glycosylated form of the protein but does not take into account the possibility that F508del-CFTR can be addressed to the PM without being fully glycosylated through nonconventional trafficking [22]. ELISA-based methods were based on a 3HA tag inserted after the Asn at position 901 (N901) in the fourth external loop of CFTR, allowing the detection of CFTR at PM independently of its glycosylation state (Supplemental Figure S4A,C). Human embryonic kidney HEK293 GripTite (HEK) cells stably expressing WT- or F508del-CFTR-3HA were previously characterized and validated [23,24]. First, we checked that WT- and F508del-CFTR-HEK293 cells did not express HspB4 or HspB5 endogenously but expressed HspB1 as in CFBE cell lines (Supplemental Figure S2). Then, to evaluate and quantify the PM localization of WT- and F508del-CFTR, we used two different ELISA-based methods on HEK293 cells stably expressing CFTR-3HA where 3HA tag was inserted after the Asn at position 901 (N901) in the fourth external loop of CFTR (Supplemental Figure S4A,C) [23,24]. Like CFBE cells, HEK293 cells did not express HspB4 or HspB5 endogenously but expressed HspB1 (Supplemental Figure S2). To validate these methods, we overexpressed HspB1 and HspB4 since they have been described to decrease F508del-CFTR PM localization [12,25]. As expected, HspB1 or HspB4 overexpression did not rescue the F508del-CFTR at PM (Figure 3). In these experiments, untransfected F508del-CFTR HEK293 cells (NT) were used as a negative control, whereas cells expressing WT-CFTR or low temperature which rescued F508del-CFTR were used as positive controls. With protein biotinylated extracts, HspB5 overexpression partially rescued F508del-CFTR at PM (21.45% ± 10.81%) compared to the low temperature rescued F508del-CFTR (+41.88% ± 8.7%) but this rescue was significant compared to control (*p* < 0.05) (Figure 3A). When measuring F508del-CFTR density at PM on live cells, HspB5 overexpression increased significantly (*p* < 0.01) the F508del-CFTR at PM (13.17% ± 2.66%) (Figure 3B).

**Figure 4:**
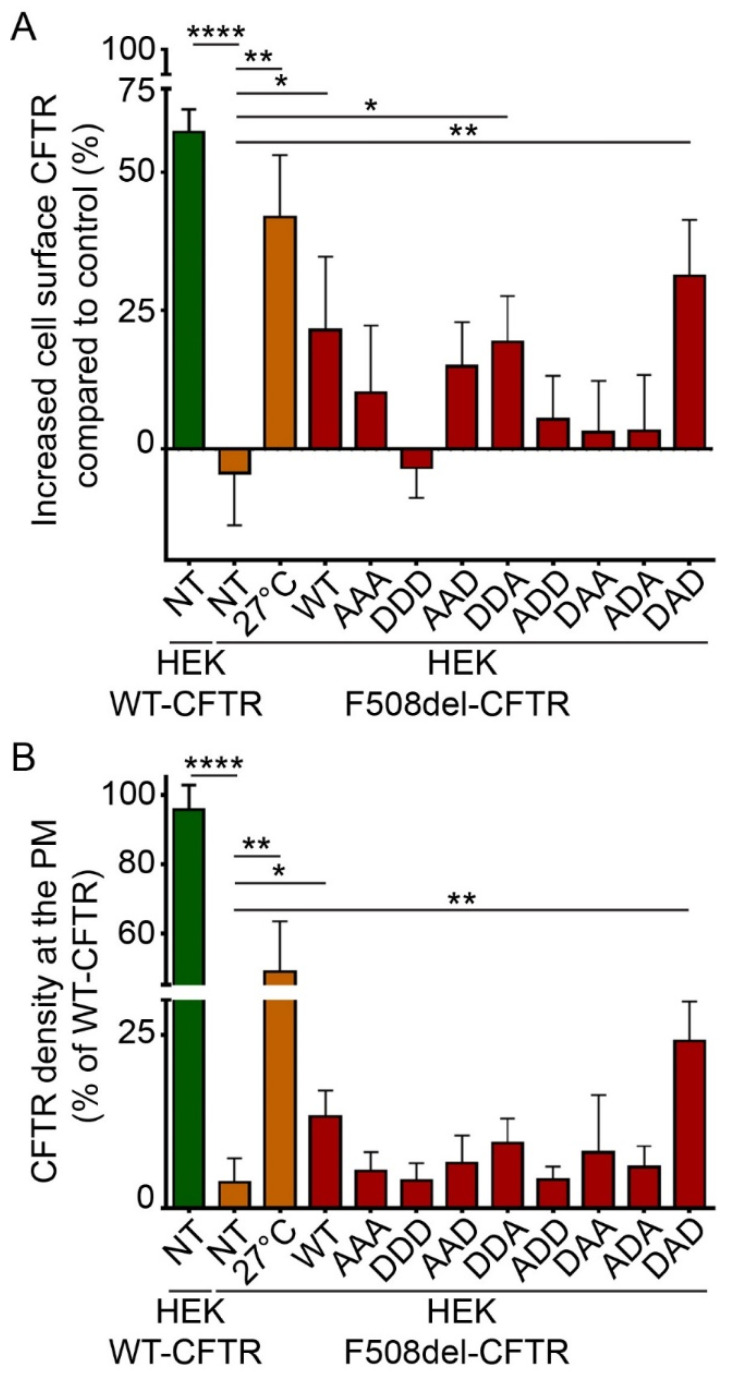
Phosphorylation-dependent ability of HspB5 to rescue the PM localization of F508del-CFTR in HEK293 GripTite cells. HEK293 GripTite (HEK) cells stably expressing F508del-CFTR-3HA were transfected with phosphomimetic mutant of HspB5 constructs. Cells were used 24 h post-transfection for detection of PM CFTR (*n* = 5). Untransfected F508del-CFTR-3HA HEK (NT) were used as a negative control. Untransfected F508del-CFTR-3HA HEK grown at 27 °C (27 °C) and untransfected WT-CFTR-3HA HEK (NT) were used as positive controls. * *p* < 0.05, ** *p* < 0.01, **** *p* < 0.0001 compared to untransfected F508del-CFTR-3HA HEK (NT). Differences were obtained using a one-way ANOVA followed by the post-hoc Dunnett test. (**A**) ELISA-based assay to measure CFTR at the PM. Each condition was analyzed in duplicate using 5 µg of protein extracts. Experiments were conducted five times. (**B**) Cell surface density measurement. Each condition was analyzed in duplicate. Experiments were conducted five times.

To pinpoint if one of the serine residues in HspB5 was involved in this rescuing effect, we tested phosphomimetic mutants of HspB5. In these mutants, serine residues are replaced by alanine (A) to mimic nonphosphorylated residues, or by aspartate (D) to mimic constitutive phosphorylation. As HspB5 contains three phosphorylatable serine residues, we constructed eight phosphomimetic mutants, as illustrated in Supplemental Figure S3. DDA- (S19D; S45D; S59A) and to a greater extent, DAD-HspB5 (S19D; S45A; S59D) mutants had the capacity to rescue the F508del-CFTR protein significantly at the PM when using biotinylated protein extracts (Figure 4A). The measurement of the F508del-CFTR density at PM in live cells confirmed that the DAD-HspB5 mutant had a better ability than WT-HspB5 to correct F508del-CFTR localization (Figure 4B).

To further confirm our results, we visualized the PM localization of F508del-CFTR by immunofluorescence experiments in BEAS-2B cells (Supplementary Figure S5A). As expected, the best F508del-CFTR PM localization was seen when DAD-HspB5 was co-expressed (Supplementary Figure S5B).

### 2.4. DAD-HspB5 Increases F508del-CFTR Stability

**Figure 5:**
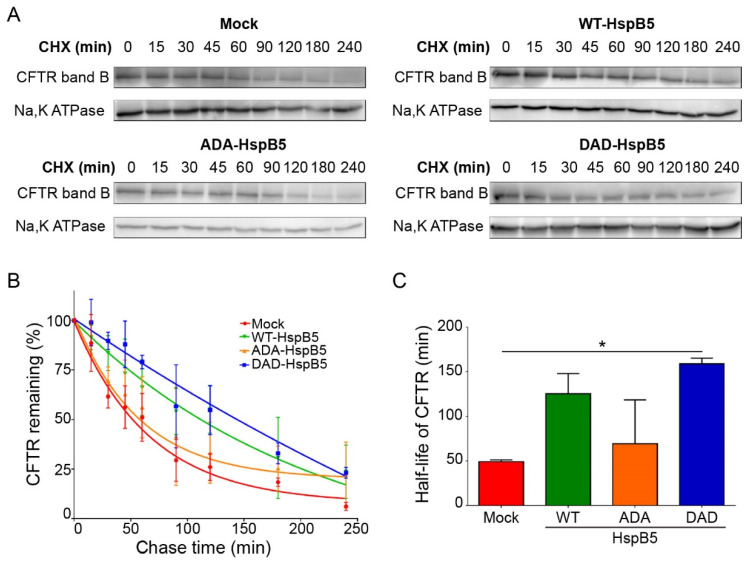
Half-life of deltaF508-CFTR in the presence of WT or phosphomimetic HspB5 in CFBE by cycloheximide (CHX)-chase experiments. Twenty-four hours after transfection, CFBE cells expressing F508del-CFTR were treated with 100 μg/mL of cycloheximide (CHX) for the indicated times and total protein extracts were analyzed. (**A**) Representative images of Western blotting showing core-glycosylated F508del-CFTR (band B). Na,K-ATPase was used to control for equal loading onto the gel. (**B**) Signal quantification of the bands. Proteins quantification were standardized to CFTR levels at t = 0 min. (*n* = 3). (**C**) Half-life of F508del-CFTR transfected with empty vector or WT-, ADA- (S19A; S45D; S59A) or DAD-HspB5 (S19D; S45A; S59D) construct. * *p* < 0.05 compared to F508del-CFTR CFBE cells transfected with an empty vector. Differences were obtained using the Kruskal–Wallis test.

We have shown that WT-HspB5 and, more specifically, DAD-HspB5 increased the amount of F508del-CFTR at PM and its activity (Figure 3 and Figure 4). As HspB5 is a member of the quality control system, we hypothesized that it could act early in the trafficking pathway and stabilize the core-glycosylated F508del-CFTR. To test this hypothesis, we performed cycloheximide (CHX)-chase experiments in F508del-CFTR CFBE transfected cells with WT-, ADA- (S19A; S45D; S59A), or DAD-HspB5 construct, or empty vector control. F508del-CFTR protein expression was analyzed on total protein extracts by immunoblot (Figure 5A). Band B of CFTR was almost undetectable after 3 h of CHX treatment in mock cells, in contrast to cells transfected with WT-, ADA-, or DAD-HspB5. Quantification of band B of F508del-CFTR (Figure 5B) showed that DAD-HspB5 stabilized band B. By calculating half-life, we confirmed that DAD-HspB5 increased the stability of core-glycosylated F508del-CFTR significantly with a half-life of 158.9 +/− 4.8 min against 49.1 +/− 1.5 min in mock condition (*p* < 0.05) (Figure 5C).

### 2.5. HspB5 and Its Phosphomimetic DAD Mutant Increase Halide Transport and Act Additively with Ivacaftor, Lumacaftor, and Orkambi Treatments

**Figure 6:**
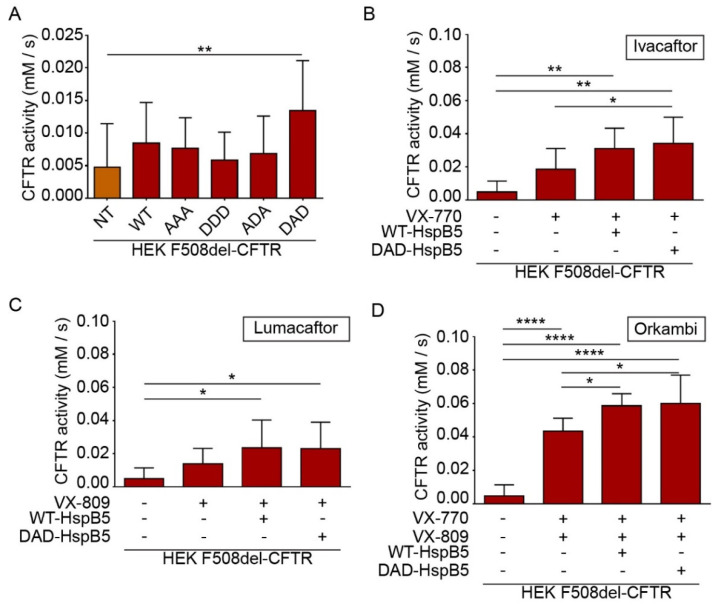
HspB5 and its phosphomimetic DAD mutant increased the F508del function and acted in synergy with Ivacaftor and Orkambi treatments. HEK293 MSR GripTite cells stably coexpressing eYFP (H148Q/I152L) and F508del-CFTR-3HA were transfected with WT- or phosphomimetic-HspB5. Before halide transport assay to measure CFTR (or its mutant) function, untransfected cells (NT) were treated with 0.1% DMSO and transfected cells were treated, or not, with VX-770 (10 µM–30 min), VX-809 (3 µM–24 h), or combination VX-770 and VX-809 (24 h). Each condition was done in triplicate, and the experiments were repeated three times. * *p* < 0.05, ** *p* < 0.01, **** *p* < 0.0001. Differences were obtained using a one-way ANOVA followed by the post-hoc Dunnett test. (**A**) Halide transport assay in F508del-CFTR-3HA-eYFP-HEK transfected WT- or phosphomimetic-HspB5. (**B**) Halide transport assay in F508del-CFTR-3HA-eYFP-HEK transfected WT- or phosphomimetic-HspB5 and treated with VX-770. (**C**) Halide transport assay in F508del-CFTR-3HA-eYFP-HEK transfected WT- or phosphomimetic-HspB5 and treated with VX-809. (**D**) Halide transport assay in F508del-CFTR-3HA-eYFP-HEK transfected WT- or phosphomimetic-HspB5 and treated with VX-770 and VX-809.

CFTR is a cyclic adenosine monophosphate (cAMP)-dependent chloride channel, and its function can be measured by a halide sensitive assay using a specifically designed H148Q/I152L (eYFP) [26]. This method is particularly useful to screen molecules of interest for CF treatment [27,28]. To determine the impact of overexpression of HspB5-WT or its phosphomimetic mutants (AAA (S19A; S45A; S59A), DDD (S19D; S45D; S59D), ADA, and DAD), we transiently transfected HEK293 GripTite cells stably coexpressing eYFP(H148Q/I152L) and F508del-CFTR-3HA [24,27,28]. The eYFP halide assay showed that DAD-HspB5 increased CFTR activity significantly (*p* < 0.01) (Figure 6A).

CF patients can be treated by FDA (US Food and Drug Administration) approved drugs Ivacaftor (VX-770), Lumacaftor (VX-809), or Orkambi (VX-770 + VX-809) but these treatments lead to varying clinical responses within the targeted cohort [29]. Research of molecules that could enhance the effect of these drugs is crucial to optimize CF treatment. Therefore, we examined the combination of these compounds with WT- and DAD-HspB5 on the F508del-CFTR function. Interestingly, we observed only DAD-HspB5 overexpression increased the effect of Ivacaftor (VX-770) (*p* < 0.05) (Figure 6B) significantly. WT- and DAD-HspB5 overexpression increased the function of F508del-CFTR significantly, when combined with Lumacaftor (VX-809) in comparison to untransfected cells treated with DMSO (*p* < 0.05) (Figure 6C). Strikingly both WT- and DAD-HspB5 increased the effect of Orkambi (VX-770 + VX-809) significantly on the F508del-CFTR function (*p* < 0.05) (Figure 6D).

## 3. Discussion

During the last decade, evidence has accumulated towards the broad beneficial activities of overexpressed HspB5 in conformational and inflammatory diseases [30,31], whereas deleterious effects were reported in cancer and idiopathic fibrosis [32,33,34]. In CF, the potential effect of this chaperone-like protein was not yet investigated. According to the literature, the first hypothesis would be that HspB5 could act as HspB1 and HspB4 by promoting the specific degradation of F508del-CFTR [12,13,14,15,25]. However, a second hypothesis emerges from what occurs with two TMPs (Frizzled 4 receptor and ATP7B) similar to the multi-spanned transmembrane CFTR where HspB5 rescues the mutated TMPs. Thus, HspB5 could rescue F508del-CFTR rather than promoting its degradation [18,19].

We first observed that endogenous expression of HspB5 was increased in HNEC derived from nasal polyps of CF and CRS patients compared to HNEC derived from nasal brushing of healthy controls. This result is consistent with earlier reports showing elevated HspB5 expression in several conformational or inflammatory diseases [35,36]. We also observed that HspB5 expression was increased in the lungs of CF mice (F508del/F508del) compared to WT littermates, suggesting that misfolding of CFTR was sufficient to induce HspB5 expression. However, following lipopolysaccharides (LPS from *P. aeruginosa*) stimulation, increased expression of HspB5 was only detectable in WT mice, revealing an incapacity of CF mice to increase the level of HspB5 in response to LPS. It could be interesting to evaluate if this defective response can be related to the unpaired inflammatory response observed in CF. We then used native CFBE and WT- or F508del-CFTR CFBE to evaluate the impact of HspB5 overexpression. In these cells, HspB5 and HspB4 expression was undetectable, whereas HspB1 was expressed. After HspB5 transfection in WT- or F508del-CFTR CFBE cells, analysis of phosphorylation status of HspB5 revealed hypophosphorylation on its serine 19 and 59 in F508del-CFTR compared to WT-CFTR CFBE cells. This result was particularly important, considering that the beneficial effect on TMPs of HspB5 was demonstrated as tightly regulated by its phosphorylation status [19]. To confirm the importance of HspB5 phosphorylation in CF, we decided to include phosphomimetic mutants in our study. First, we determined the impact of HspB5 and phosphomimetic mutants’ overexpression on F508del-CFTR localization by two different ELISA-based methods and immunofluorescence confocal microscopy. We established that contrary to HspB1 or HspB4, HspB5 increased the PM localization of F508del-CFTR significantly, and this capacity to restore CFTR localization was regulated by its phosphorylation. Interestingly, one of the phosphomimetic mutants (S19D; S45A; S59D, i.e., DAD) had the highest ability to restore F508del-CFTR at the PM. We established that overexpression of this phosphomimetic HspB5 mutant increased the activity (measured by halide transport) of F508del-CFTR significantly. This activity was further enhanced in the presence of Ivacaftor (VX-770) or Orkambi (VX-770 and VX-809) treatments. One hypothesis to explain this additive effect could be that HspB5 has a dominant effect on endogenous HspB1 by reducing its binding to F508del-CFTR, which would allow it to escape degradation. This hypothesis is reinforced by the fact that CHX-pulse-chase experiments in F508del-CFTR CFBE cells revealed that HspB5 increased the half-life of the core-glycosylated form of F508del-CFTR significantly. Further studies would be needed to establish whether HspB5 rescues F508del-CFTR at PM by using the conventional or unconventional secretion pathway. To this aim, the overexpression of HspB5 in primary cells would give a more physiological statement.

Although our study focused on the impact of HspB5 overexpression and its phosphorylation on F508del-CFTR trafficking, stability, and function, HspB5 exhibited many other functions of interest for CF treatment. One of the most interesting effects of HspB5 was its role in inflammation since, in CF, inflammation plays a critical role in disease progression and lung pathology. Screening of molecules able to decrease CF chronic inflammation is an active area of research [37]. Recently, Xu et al. [38] reported that HspB5 mRNA expression was decreased in inflamed mucosa of inflammatory bowel disease patients compared to noninflamed specimens. Interestingly, TAT-HspB5 recombinant protein administration alleviated intestinal mucosal inflammation in mice. This study also revealed that the transfection of HspB5 inhibited the production of several proinflammatory cytokines secreted by LPS-induced macrophages. The anti-inflammatory property linked to the administration of HspB5 has also been demonstrated in a mouse model of chronic obstruction pulmonary disease induced by cigarette smoke [39]. In this model, the administration of HspB5 was done using HspB5-loaded PLGA microparticles. They demonstrated a reduction in lung inflammation and activation of the immune-regulatory macrophage response. The most advanced use of exogenous HspB5 administration as a treatment is a phase IIa trial in Multiple Sclerosis, which shows that HspB5 treatment is safe, well-tolerated, and provides clinical benefits for the patients. [40]. One other interesting effect is the impact of HspB5 during infection. Recently, it has been reported that HspB5 overexpression in the heart significantly attenuated coxsackieviral infection by decreasing the viral protein production, suggesting an antiviral function for HspB5 [41]. Viral respiratory infections contribute to chronic bacterial colonization of CF lungs. Following infection with the Coxsackie virus B3, CF mice compared to WT mice had increased morbidity and mortality because of an impaired viral clearance [42]. Such an antiviral effect of HspB5 on CF would be worth testing in the future. 

Our study highlights that HspB5 is the first sHsps which had a positive effect on improving CF disease, and further studies should evaluate the potential of HspB5 for CF treatment.

## 4. Material and Methods

### 4.1. Cell Lines

Human bronchial epithelial cell line CFBE41o- (ref: SCC151, Sigma Aldrich, St. Louis, MO, USA) derived from a CF patient were modified to stably expressing wild-type CFTR (WT-CFTR) and F508del CFTR (F508del-CFTR) [43,44,45]. CFBE cells were obtained and used following a special agreement with University of California San Francisco (UCSF). CFBE cells were grown in MEM (Life Technologies, Carlsbad, CA, USA) supplemented with 10% FBS (Eurobio Scientific, France), 1% penicillin/streptomycin (P/S) (Life Technologies, Carlsbad, CA, USA) and 1% L-glutamine (Life Technologies, Carlsbad, CA, USA).

Human embryonic kidney expressing stably macrophage scavenger receptor (HEK293 MSR GripTite; ref: R79507, Thermo Fisher, Waltham, MA, USA) cell line modified to stably expressing wild-type CFTR-3HA or F508del-CFTR-3HA (the HA-tag is localized within the extracellular loop of CFTR) [24] were grown in DMEM (Life Technologies, Carlsbad, CA, USA) containing 10% FBS, 1% P/S, 0.6 mg/mL G418, and 10 µg/mL blasticidin.

Human embryonic kidney (HEK293 MSR GripTite) cells stably coexpressing eYFP (H148Q/I152L) and WT- or F508del-CFTR-3HA [24] were grown in DMEM supplemented with 10% FBS, 50 µg/mL zeocin, 0.6 mg/mL G418, 10 µg/mL blasticidin.

Human bronchial epithelium cell line BEAS-2B (ref: CRL-9609™, ATCC^®^, Manassas, VA, USA) were cultured in LHC-8 medium (Life Technologies, Carlsbad, CA, USA) supplemented with 10% FBS, 1% L-glutamine, and 1% P/S.

All cell lines were cultured at 37 °C, 5% CO_2_, in a humidified atmosphere.

### 4.2. Human Nasal Epithelial Cells

We included 20 patients from 3 different groups: (*i*) healthy volunteers with no history of sino-pulmonary disease, no mutation detected by complete scanning of the coding sequences, or large rearrangements following our routine analysis on gDNA to look for *CFTR* gene mutations (*n* = 3). (*ii*) CF patients with nasal polyps (*n* = 4) (*iii*) non CF patients with chronic rhinosinusitis (CRS) with nasal polyps (*n* = 13). Details on patients are given in Supplementary Table S1. All patients were followed in the ENT Department in the Centre Hospitalier Intercommunal of Créteil (CHIC), and informed consent was obtained. Healthy volunteers have been subject to a nasal brushing and blood sample (for an extensive study of the *CFTR* gene) was done. The protocol was approved by our ethics committee (Comité de Protection des Personnes) Ile de France XII in March 2011.

During ENT consultation nasal brushing have been practiced under nasal endoscopy, after local anesthesia with a cotton pellet soaked in lidocaine (3.4%), HNEC from healthy subjects were collected from nasal epithelium by brushing inferior turbinates. HNEC from polyps from patients with CRS or with CF were collected during surgery (ethmoidectomy) and immediately transferred to the laboratory in DMEM/F12 supplemented with antibiotics (100 U/mL of penicillin, 100 mg/mL of streptomycin, 2.5 µg/mL of amphotericin B and 100 mg/mL of gentamicin). After centrifugation (1000 rpm, 10 min), the cell pellet is collected and suspended in DMEM/F12 (3 mL), with antibiotics (100 U/mL of penicillin, 100 mg/mL of streptomycin, 2.5 µg/mL of amphotericin B and 100 mg/mL of gentamicin) and 5% fetal calf serum. Finally, HNEC were plated on permeable polycarbonate supports (Transwell^®^, Costar, Cambridge, USA) (1.10^6^ cells/cm^2^). All inserts had a diameter of 6.5 mM and were coated with type IV collagen. HNEC were incubated at 37 °C in 5% CO_2_. For the first 24 h, HNEC were incubated with 1 mL of DMEM/F12-antibiotics with 2% Ultroser G outside the insert. After 24–36 h, medium was removed inside the inserts to place the cells at an air–liquid interface, medium outside the inserts was then changed daily. Studies were performed on day 15.

### 4.3. Mouse Lung Samples

Mouse lung samples were collected in a previous study [46]. All animals care and experimental protocols complied with INSERM guidelines and were approved by the Regional Ethical Committee C2EA-16 (n°12.074-11/12/12-17). The experimental procedures used in the work were as humane as possible. We used lung tissues from three CF male mice (11–16 weeks old, average weight 23 to 24 g) homozygous for the F508del-CFTR mutation (F508del/F508del), cftrtm^1Eur^ [47] and three normal homozygous WT littermates (+/+) (average weight 27 to 28 g). Samples were stocked at −80 °C before extraction of protein content.

Half of the lung were used for protein extraction with 500 µL of lysis buffer (50 mM Tris pH7.4, 1% Nonidet P40, 2% Glycerol, 136 mM NaCl, 1 mM EGTA pH8, 10 mM NaF, plus complete protease inhibitor cocktail tablets (Roche, Switzerland)). Tissues were homogenized with a potter and centrifugated 15 min at 15,000× *g* at 4 °C to remove cellular debris. Then, supernatants were separated in 5 aliquots, and total protein extract concentration was measured using the DCTM kit (Bio-Rad, Hercules, CA, USA).

### 4.4. Vector Constructs and Transfection

Cells were seeded at 80% confluence and transfected using Lipofectamine 2000 (Invitrogen, Carlsbad, CA, USA) or Polyjet DNA in vitro transfection reagent (Signagen, Frederick, MD, USA) according to the manufacturer’s instructions. Empty vector pcDNA3 (Invitrogen, Carlsbad, CA, USA) was used as control. Cloning of HspB5 cDNA into pcDNA3 has been described previously [21]. The phosphorylation mimicking mutant HspB5 (AAA, DAA, ADA, AAD, DDA, AAD, DAD, DDD) were obtained by site-directed mutagenesis using Quik Change XL Site-Directed Mutagenesis Kit (Agilent, Santa Clara, CA, USA) according to the manufacturer’s instructions. A list of the primers used is given Supplementary Table S2. All constructs were verified by sequencing. Cloning of HspB1 cDNA into pcDNA3 and HspB4 cDNA into FRT vector have been described previously [48,49]. pcDNA3-HspB1 and Frt-hspB4 were gifts from André-Patrick Arrigo and Harm Kampinga (Addgene plasmid # 63095, Watertown, NY, USA), respectively. The cloning details of CFTR cDNA into pTRACER-CMV (Invitrogen, Carlsbad, CA, USA) and obtention of F508del-CFTR mutant has been described previously [50].

### 4.5. Immunofluorescence Confocal Microscopy

BEAS-2B were grown on coverslips, transfected, and 24 h later were washed twice with ice-cold PBS, fixed and permeabilized with cold methanol:acetone at a ratio of 7:3 for 10 min at 4 °C. Subsequently, cells were incubated with 5% FBS and 5% BSA in PBS for 1h at room temperature and after being washed twice with ice-cold PBS, incubated with primary monoclonal mouse antibody (Ab) against CFTR (clone 13.1 MAB1660, R&D Systems, Minneapolis, MN, USA) and polyclonal rabbit Ab against HspB5 (8851, Cell Signaling, Danvers, MA, USA) overnight at 4 °C, at 1:50 and 1:150 dilution, respectively; washed with PBS, and incubated in the dark with secondary goat anti-mouse Ab conjugated to Alexa Fluor 568 (Life Technologies) and goat anti-rabbit Ab conjugated to Alexa Fluor 488 (Life Technologies, Carlsbad, CA, USA) at 1:75 dilution for 1 h at room temperature; washed with PBS and incubated in the dark with To-Pro-3 Iodide (Life Technologies, Carlsbad, CA, USA) at 1:5000 dilution for 15 min at room temperature; then washed with PBS. Coverslips were mounted on slides using Vectashield mounting medium for fluorescence (Vector Laboratories, Burlingame, CA, USA). Confocal acquisitions were performed with a Zeiss confocal LSM 510 and the Zeiss Zen 2009 software. Fluorescence was collected with a 63×/1.4 Numerical Aperture lens. The appropriate laser and fluorescence filter sets were fitted to optimize data acquisition and limit bleed-through and crosstalk. At least 10 representative images were taken by condition, and each experiment was repeat 3 times.

### 4.6. Total Cellular Protein Extraction and Western-Blotting

Twenty-four hours after transfection, cells were harvested and lysed in RIPA 1X lysis buffer (50 mM Tris-HCl pH 7.4, 1% NP-40, 0.5% Na-deoxycholate, 0.1% SDS, 150 mM NaCl, 2 mM EDTA, 50 mM NaF) plus complete protease inhibitor cocktail tablets (Roche, Switzerland). Extracts were vortexed for 30 sec and centrifuged to remove cellular debris. Total protein extract concentration was measured using the DCTM kit (Bio-Rad, Hercules, CA, USA). Equal amounts of protein (30 μg) were solubilized in Laemmli sample buffer 5X. The samples were then migrated on 7% or 15% sodium dodecyl sulfate polyacrylamide gel electrophoresis (SDS-PAGE) and then transferred onto a nitrocellulose membrane (GE Healthcare, Chicago, IL, USA). Membranes were blocked for 1 h at room temperature with blocking buffer (TBS 1X + 0.05% Tween-20 + 5% milk or BSA free of IgG). Membranes were probed overnight at 4 °C with specific primary pAb against HspB5 (8851, Cell Signaling, Danvers, MA, USA), pAb against [pSer19] αB-crystallin (ADI-SPA-225, Enzo Life Sciences, France), pAb against [pSer45] αB-crystallin (ADI-SPA-226, Enzo Life Sciences, France), pAb against [pSer59] αB-crystallin (ADI-SPA-227, Enzo Life Sciences, France), mAb against Hsp27/HspB1 (2402, Cell Signaling, Danvers, MA, USA), pAb against CRYAA/HspB4 (HPA038430, Sigma, St. Louis, MO, USA), mAb against CFTR 570 and 596 (from Antibody Distribution Program of Cystic Fibrosis Foundation),pAb against β-Actin (Cell Signaling, Danvers, MA, USA) and pAb against Na,K-ATPase (Sigma, St. Louis, MO, USA) antibodies, diluted to 1:2000 in TBS 1X + 0.05% Tween-20 + 0.2% BSA. The membranes were probed with anti-mouse or anti-rabbit IgG HPR-linked antibodies (Cell Signaling Technology, Danvers, MA, USA), diluted to 1:50,000 or 1:10,000 respectively in TBS 1X + 0.05% Tween-20 + 0.2% BSA, for 1 h at room temperature. Signals were then revealed using the ECL Prime Western Blotting Detection Reagent kit (GE Healthcare, Chicago, IL, USA), and images were acquired in a dark room using a G:Box (Syngene, India) equipped with GeneSys software. Western blot analysis and quantification used Gene Tools (Syngene, India). Protein quantification was normalized to β-Actin or Na,K-ATPase. The degree of phosphorylation of the various HspB5 species was determined as previously [21].

### 4.7. HspB5 ELISA Assay

HspB5 ELISA Assays were performed in 96-well high-binding polystyrene microtiter plates (Enzo Life Science, France) using the ImmunoSet αB-Crystallin ELISA development set (ADI-960-074 Enzo Life Science, France) according to the manufacturer’s instructions. Equal amounts of protein from HNEC or mice’s lungs were used (2.5 µg). Each sample (*n* = 3 per condition) was tested in triplicate.

### 4.8. ELISA-based Assay to Measure CFTR at the PM

HEK293 MSR GripTite cells stably expressing WT- or F508del-CFTR-3HA were transfected with WT- or phosphomimetic-HspB5 or empty construct in 12-well plate. Twenty-four hours after transfection, cells were biotinylated with 0.5 mg/mL sulfo-NHS-LC-Biotin in PBS for 15 min, then washed with ice-cold PBS and lysed with RIPA 1X containing anti-protease. To capture WT- or F508del-CFTR, 5 μg of total lysate protein (per well) were incubated with anti-HA antibody (1:1000) in 96-well plate for 2 h at 4 °C. After 3 washes with PBST (PBS + 0.05% Tween), SA-HRP at 1:1000 in ELISA buffer (PBST + 0.5% BSA) was added during 20 min. After 3 washing, the plates were developed with TMB substrate, the reaction was stopped with 1 N H_2_SO_4_ and were read at 450 nm. Details on the method and the controls used are given in Supplementary Figure S4A,B.

### 4.9. Cell Surface Density Measurement of CFTR-3HA

HEK293 MSR GripTite cells stably expressing WT- or F508del-CFTR-3HA were transfected with WT- or phosphomimetic-HspB5 or empty construct in 12-well plate. Twenty-four-hour post-transfection, cells were incubated in blocking buffer (ice-cold PBS, 0.5% BSA) for 20 min. Then, cells were incubated with anti-HA antibody at 1:1000 in for 1 h at 4 °C. After 3 washes with ice-cold PBS, HRP-conjugated anti-mouse IgG (1:1000) were incubated for 1h at 4 °C. After 6 washes with ice-cold PBS, 200 μL of AmplexR Red reaction mix (Thermo Fischer Scientific, Waltham, MA, USA) was added for 10 to 20 min in the dark. The mix was transfer in a black 96-well plated, and the signal was read at 544 nm excitation and 590 nm emission wavelength. The fluorescence signal was normalized with the protein concentration of the respective well to obtain the relative cell-surface CFTR-3HA density. Details on the method and the controls used are given in Supplementary Figure S4C,D.

### 4.10. YFP Halide-Exchange-Assay

HEK293 GripTite cells stably coexpressing eYFP(H148Q/I152L) and WT- or F508del-CFTR-3HA were transfected with WT- or phosphomimetic-HspB5 or empty construct in 6-well plates. Twenty-four-hours later, cells were transferred into 96-well black/clear bottom microplates. After 6 h, cells were treated with Lumacaftor (VX-809 at 3 µM) or DMSO, or untreated. The CFTR functional assay was carried out 48 h after transfection. Cells were treated or not with Ivacaftor (VX-770 at 10 µM) 30 min before the assay. Cells were incubated for 30 min with 100 μL of PBS containing cpt-AMPc (100 μM) before being transferred to a TriStar plate reader (Berthold, France). Cell fluorescence (excitation: 485 nm; emission: 535 nm) was continuously measured before and after the addition of 100 μL of PBS-NaI. Cell fluorescence recordings were normalized to the initial average value measured before the addition of PBS-NaI. To calculate the activity of CFTR, we used 3 separate wells for each group and used an exponential function which fit to the signal decay (GraphPad Software v6.0.3, San Diego, CA, USA). Representative graphs illustrating the use of an exponential function which fit to the signal decay (one phase decay) are presented in Supplementary Figure S6. The maximal slope, which corresponded to the initial influx of I^−^ into the cells, was derived. Finally, the results were converted to rates of variation in intracellular I^−^ concentration (mM/s) according to the following equation: d[I^−^]/d*t* = K*_I_*[d(*F*/*F0*)d*t*] (with K*_I_*: affinity constant of YFP for I^−^; *F*/*F_0_*: ratio of the cell fluorescence at a time versus the initial fluorescence) [51].

### 4.11. Pulse (CHX)-Chase

Twenty-four hours after transfection, CFBE cells expressing F508del-CFTR were treated with 100 µg/mL of cycloheximide (CHX) for 15, 30, 45, 60, 90, 120, 180, or 240 min. Then, after proteins extraction with RIPA 1X were analyzed by Western blotting, as described previously, using mAb against CFTR 570 and 596 (from the Antibody Distribution Program of the Cystic Fibrosis Foundation), and pAb against Na,K-ATPase (Sigma, St. Louis, MO, USA) antibodies. Signal quantification used Gene Tools (Syngene, India). Proteins quantification were normalized to Na,K-ATPase levels and were standardized to CFTR levels at t = 0 min (*n* = 3). The half-life was obtained using an exponential function (one phase decay with robust fit) which fit to the signal decay with GraphPad Prism v6.0.3 (San Diego, CA, USA).

### 4.12. Statistical Analysis

Data are presented as the mean ± SEM of at least 3 independent experiments. The normality of the distribution was tested by a Kolmogorov–Smirnov normality test. The statistical significance of differences means was assessed using a one-way Analysis of Variance (ANOVA) followed by the post-hoc test Dunnett multiple comparisons for normally distributed data. Nonparametric Kruskal–Wallis test (*n* = 3) with at least *p* < 0.05 considered as statistically significant using GraphPad Prism v6.0.3 (San Diego, CA, USA) was used for non-Gaussian or (*n* = 3) data.

## Supplementary Materials

Supplementary Materials can be found at https://www.mdpi.com/1422-0067/21/14/4844/s1.
