# Supplementary material for: Phosphorylation of the Chaperone-Like HspB5 Rescues Trafficking and Function of F508del-CFTR
Source: Int J Mol Sci. 2020 Jul 8;21(14):4844. doi: 10.3390/ijms21144844 (PMC7402320; doi:10.3390/ijms21144844)
Supplement: Supplementary file 1 [file ijms-21-04844-s001.pdf]

Supplementary Materials

Phosphorylation of the Chaperone-Like HspB5 Rescues Trafficking and Function of F508del-CFTR

Fanny Degrugillier [1], Abdel Aissat [1,2], Virginie Prulière-Escabasse [1,3], Lucie Bizard [1], Benjamin Simonneau [1], Xavier Decrouy [1], Chong Jiang [4], Daniela Rotin [4], Pascale Fanen [1,2] and Stéphanie Simon [1,*]

[1] Univ Paris Est Creteil, INSERM, IMRB, F-94010 Creteil, France; fanny.degrugillier@inserm.fr (F.D.); abdel.aissat@inserm.fr (A.A.); virginie.pruliere@inserm.fr (V.P.-E.); lucie.bizard@chicreteil.fr (L.B.); benjamin.simonneau@inserm.fr (B.S.); xavier.decrouy@inserm.fr (X.D.); pascale.fanen@inserm.fr (P.F.)

[2] AP-HP, Hôpital Henri Mondor, Département de Génétique, F-94010 Creteil, France

[3] Centre Hospitalier Intercommunal de Creteil, Service d'ORL et de Chirurgie Cervico-Faciale, F-94010 Creteil, France

[4] The Hospital for Sick Children and the University of Toronto, Toronto, ON M5G 0A4, Canada; chong.jiang@utoronto.ca (C.J.); drotin@sickkids.ca (D.R.)

* Correspondence: stephanie.simon@inserm.fr; Tel.: +33-1-49-81-68-55

Supplementary Table S1. Details on patients used in this study.

Number	Age at Surgery (years)	Sexe	Phenotype	CFTR Status	[HspB5] (ng/µg of proteins)
**M1**	7	Male	Cystic Fibrosis	F508del/ F508del	3.12
**M2**	28	Female	Cystic Fibrosis	F508del/ F508del	3.98
**M3**	19	Female	Cystic Fibrosis	F508del/ F508del	4.91
**M4**	17	Male	Cystic Fibrosis	F508del/ F508del	4.01
**PNS1**	28	Female	Chronic rhinosinusitis	-/-	3.69
**PNS2**	41	Female	Chronic rhinosinusitis	-/-	3.55
**PNS3**	61	Female	Chronic rhinosinusitis	-/-	5.87
**PNS4**	34	Female	Chronic rhinosinusitis	-/-	2.1
**PNS5**	40	Female	Chronic rhinosinusitis	-/-	2.45
**PNS6**	38	Male	Chronic rhinosinusitis	-/-	2.09
**PNS7**	61	Male	Chronic rhinosinusitis	-/-	1.99
**PNS8**	44	Male	Chronic rhinosinusitis	-/-	1.77
**PNS9**	56	Male	Chronic rhinosinusitis	-/-	5.05
**PNS10**	67	Male	Chronic rhinosinusitis	-/-	4.79
**PNS11**	52	Male	Chronic rhinosinusitis	-/-	4.59
**PNS12**	43	Female	Chronic rhinosinusitis	-/-	5.95
**PNS13**	55	Male	Chronic rhinosinusitis	-/-	4.80
**T1**	26	Female	Healthy	-/-	1.76
**T2**	31	Female	Healthy	-/-	1.71
**T3**	46	Male	Healthy	-/-	1.24

Supplementary Table S2. Primers used to construct phosphorylation mimicking mutants.

Mutation in HspB5 Protein Sequence	Primers used (5′-3′)
**S19A**	5'-aggcggctgggggcgtggaaaggaaag-3'<br>5'-ctttcctttccacgcccccagccgcct-3'
**S19D**	5'-caaagaggcggctggggtcgtggaaaggaaagaagg-3'<br>5'-ccttctttcctttccacgaccccagccgcctctttg-3'
**S45A**	5'-ccgaaggtagaagggagccagggaagtagacgtc-3'<br>5'-gacgtctacttccctggctcccttctaccttcgg-3'
**S45D**	5'-gccgaaggtagaagggatccagggaagtagacgtcg-3'<br>5'-cgacgtctacttccctggatcccttctaccttcggc-3'
**S59A**	5'-cagtgtcaaaccaggcgggtgcccgcagg-3'<br>5'-cctgcgggcacccgcctggtttgacactg-3'
**S59D**	5'-ccagtgtcaaaccagtcgggtgcccgcaggaa-3'<br>5'-ttcctgcgggcacccgactggtttgacactgg-3'

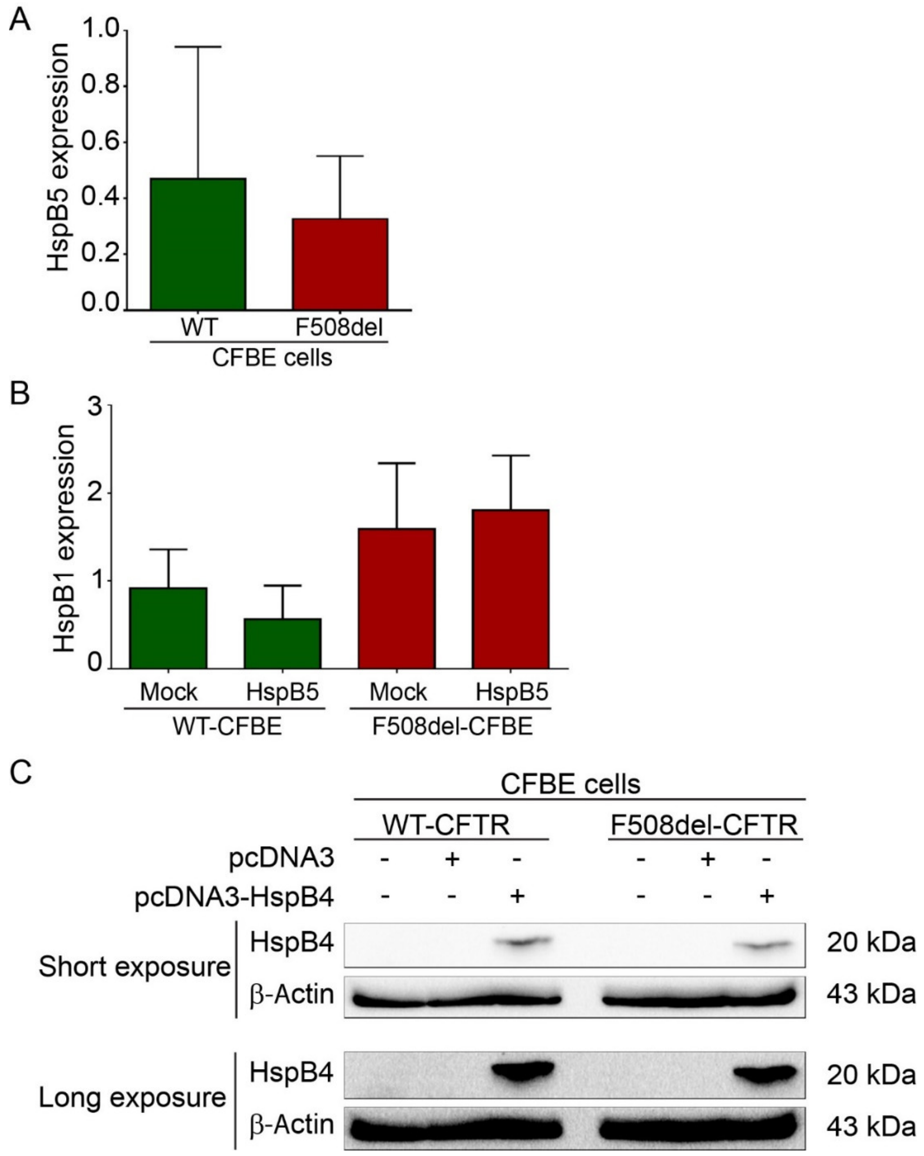

Supplementary Figure S1. (A): Evaluation of HspB5 expression in CFBE stably expressing WT- or F508el-CFTR transfected with HspB5 construct. Cells were harvested 24h after transfection and processed for SDS-PAGE/Western blotting using anti-HspB5 and β-Actin antibodies (n = 3). Quantification was done using Gene Tools software and β-actin signal was used to normalize the loading. Statistical analysis did not reveal significant difference between the samples. **(B):** Evaluation of endogenous HspB1 expression in CFBE cells stably expressing WT- or F508el-CFTR transfected or not with HspB5. Cells were transfected with empty vector or HspB5 construct and processed as in (**A**), except an anti-HspB1 antibody was used (n = 3). Quantification and statistical analysis did not reveal significant difference between the samples. **(C):** Endogenous expression of HspB4 in CFBE stably expressing WT- or F508el-CFTR. CFBE stably expressing WT- or F508del-CFTR were transfected or not with empty vector or HspB4 construct. Cells were harvested 24h after transfection and processed for SDS-PAGE/Western blotting using anti-HspB4 antibodies. Equal loading was verified using anti-β-Actin antibody. Non transfected cells were used as negative control. Images are shown at short and long exposure time (n = 3).

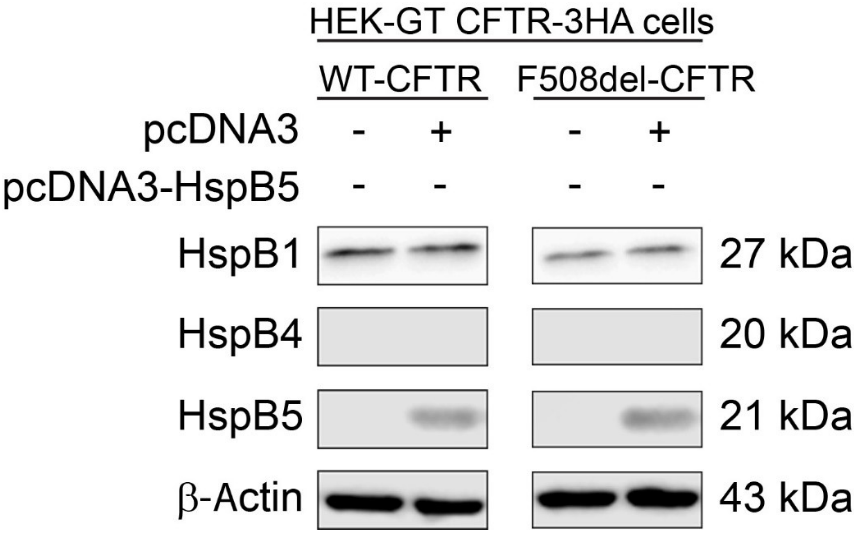

Supplementary Figure S2. Evaluation of HspB1, HspB4 and HspB5 expression in HEK293 MSR GT cells stably expressing WT- or F508del-CFTR-3HA transfected with empty vector or HspB5 construct. Cells were harvested 24 h after transfection and processed for SDS-PAGE/Western blotting using anti-HspB1, anti-HspB4, anti-HspB5 and β-Actin antibodies.

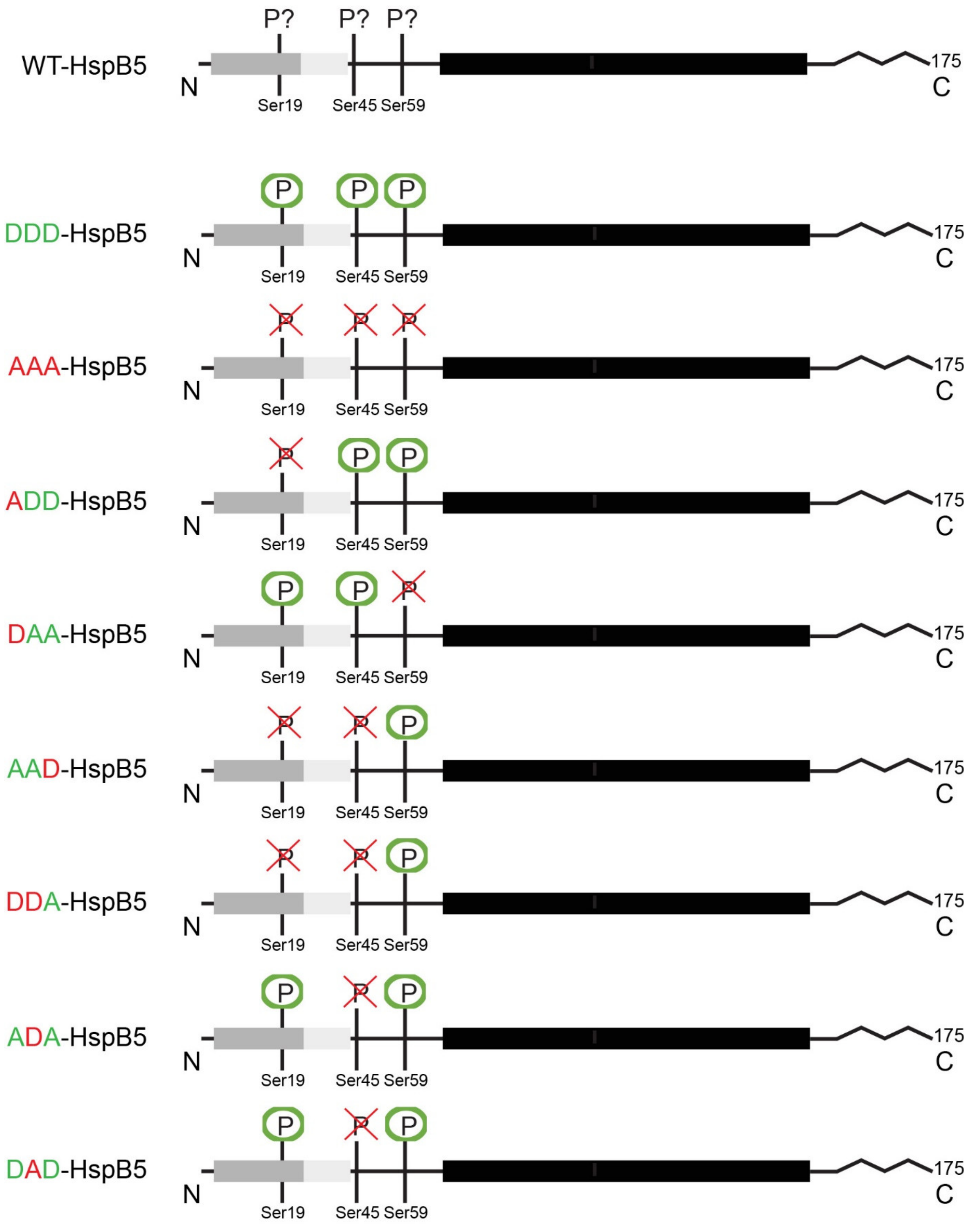

Supplementary Figure S3. Schematic representation of HspB5 phospho-mimetic mutants. Human HspB5 protein sequence contains: a conserved N-terminal region (gray box); a WDPF domain (light box); a crystallin domain (black box) and a flexible domain (VVV). HspB5 comprises 3 phosphorylatable serine sites in position 19, 45 and 59. Replacement of serine (Ser) by alanine (A) mimics the absence of phosphorylation on that site. Replacement of serine (S) by aspartate (D) mimics the phosphorylation on that site.

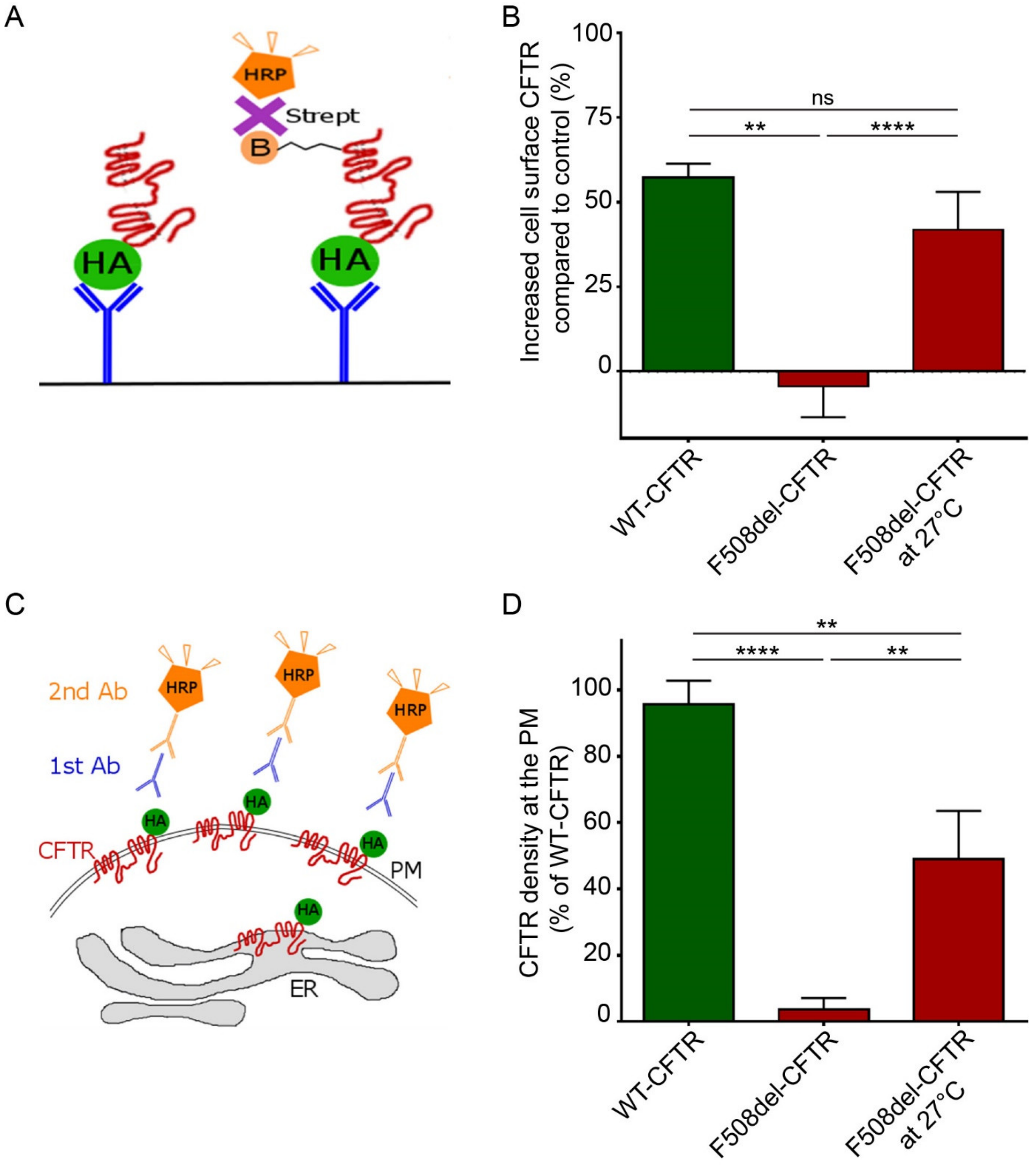

Supplementary Figure S4. Details on methodology to detect CFTR at the PM. (**A**): Principle of the ELISA-based assay to measure CFTR at the PM. Anti-HA antibodies coated on 96-well plate capture biotinylated CFTR-3HA. Plates were developed with TMB substrate. (**B**): Validation of the ELISA-based assay to measure CFTR at the PM by using total cell extracts of HEK293 stably expressing WT-CFTR or F508del-CFTR incubated (or not) at 27°C. (**C**): Principle of cell surface density measurement assay. (**D**): Validation of the cell surface density measurement of WT- or F508del-CFTR. HEK293 stably expressing WT-CFTR or F508del-CFTR incubated (or not) at 27°C.

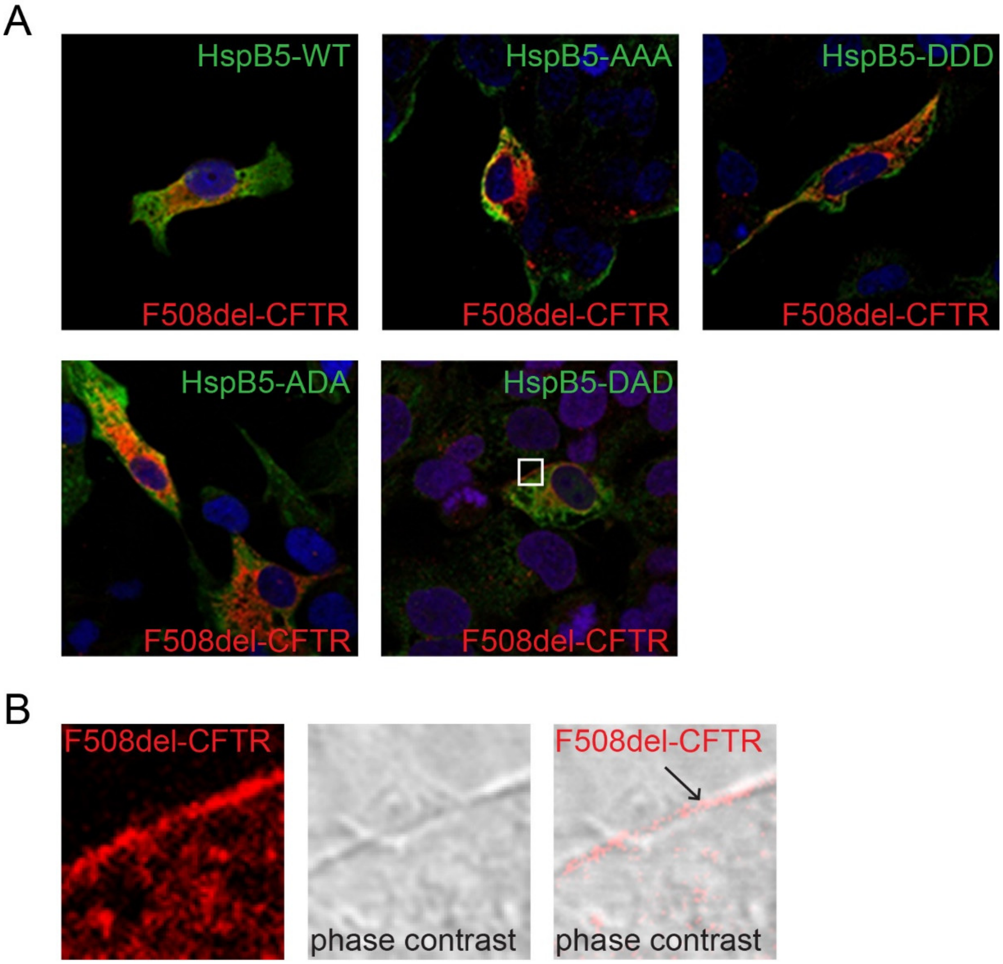

Supplementary Figure S5. Immunofluorescence confocal microscopy showing the localization of CFTR (red) and HspB5 (green). **(A):** Immunofluorescence was performed 24 h post-transfection in BEAS-2B cells (*n* = 3). Nuclei (blue) were marked using To-Pro-3 Iodide. The white box shows the cropped region used in B panel. **(B):** Cropped images from (**A**) illustrates the PM location of F508del-CFTR in BEAS-2B cells transfected with DAD-HspB5. Black arrow highlights the localization at the PM of F508del-CFTR in presence of DAD-HspB5.

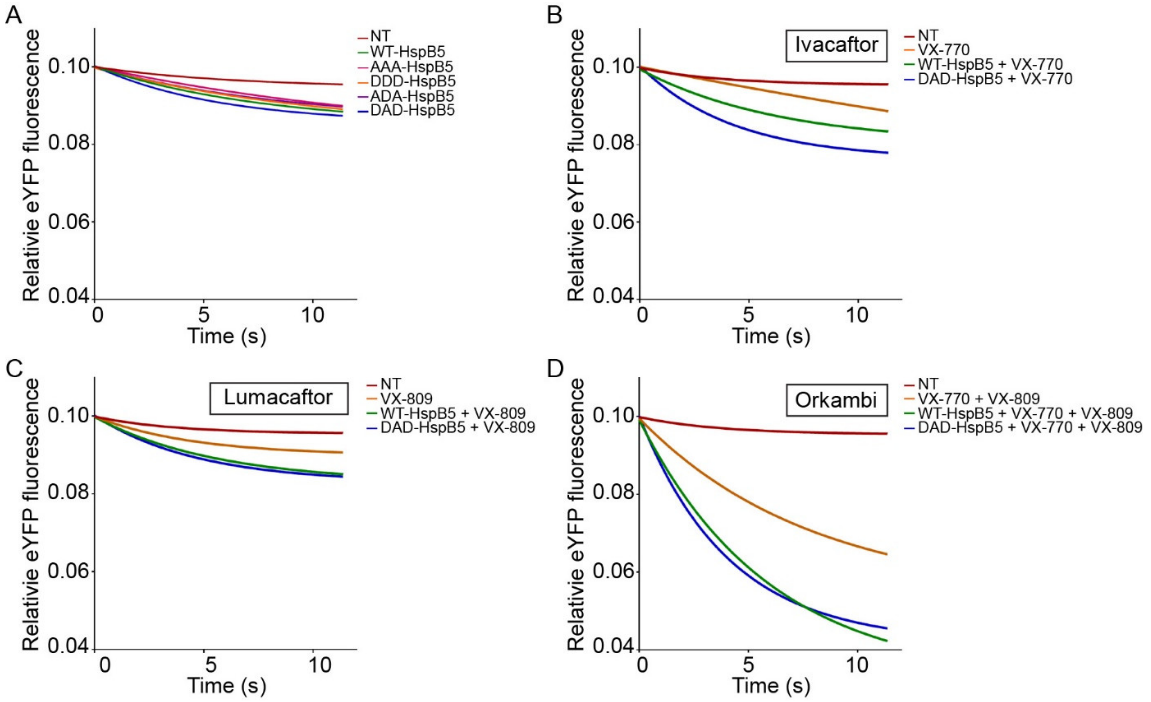

Supplementary Figure S6. Representative curves illustrating the use of exponential function which fit to the signal decay (one phase decay) for YFP Halide-Exchange-Assay. (**A**): Curves corresponding to Figure 6A. (**B**): Curves corresponding to Figure 6B. (**C**): Curves corresponding to Figure 6C. (**D**): Curves corresponding to Figure 6D.
